# Synthesis, dielectric, magnetic, and photoluminescence properties of two new hybrid rare-earth double perovskites

**DOI:** 10.3389/fchem.2022.969156

**Published:** 2022-08-05

**Authors:** Ze-Jie Wang, Long-He Li, Yan Feng, Qin-Wen Wang, Ling-Kun Wu, Jian-Rong Li, Heng-Yun Ye

**Affiliations:** Chaotic Matter Science Research Center, Jiangxi University of Science and Technology, Ganzhou, China

**Keywords:** organic–inorganic hybrid, rare earths, double perovskites, fluorescence, magnetic

## Abstract

Two new organic–inorganic hybrid double perovskites (R3HQ)_4_CsSm(NO_3_)_8_ (**1**) (R3HQ = (*R*)-(-)-3-quinuclidinol) and (R3HQ)_4_CsEu(NO_3_)_8_ (**2**) were synthesized and characterized. Compounds **1** and **2** exhibit obvious phase transitions at 379 and 375 K, respectively, confirmed by differential scanning calorimetry (DSC) and variable temperature powder X-ray diffraction. The rapid switching between high- and low-dielectric states makes it a typical dielectric material with a switchable dielectric constant for thermal stimulus response. Furthermore, **1** and **2** show attractive photoluminescence and paramagnetic behavior, and the fluorescence quantum yield of **2** reached 14.6%. These results show that compounds **1** and **2** can be used as excellent candidates for multifunctional intelligent materials, which also provides a new way for development of multifunctional materials.

## Graphical Abstract

Two new organic-inorganic hybrid rare-earth double perovskites exhibit interesting properties such as photoluminescence, magnetism, and structural phase transition.
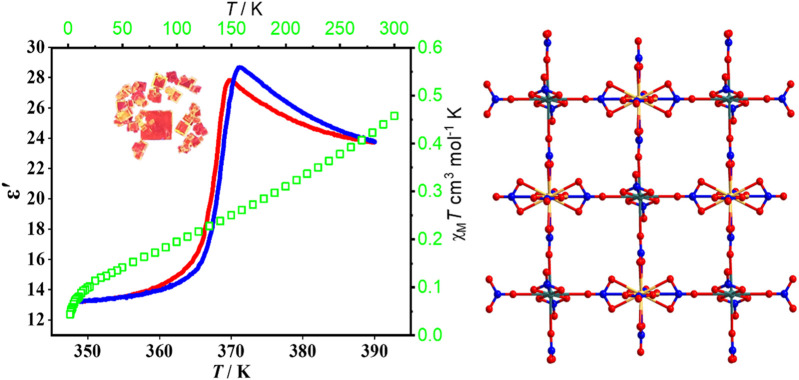

## Introduction

Perovskite structure, typically of ABX_3_-type perovskites, comprises a three-dimensional (3D) network by corner-sharing BX_6_ octahedra, where the B atom is a metal cation and the A-site cation is located in the octahedra center (Wang et al., 2021; Zhou et al., 2021; Xu et al., 2022). For ∼20 years, extensive interest has been focused on organic–inorganic hybrid perovskites (OIHPs) owing to their potential applications in areas such as ferroelectric, piezoelectric, and photovoltaic, etc. (Saparov and Mitzi, 2016; Chen et al., 2019; Shi et al., 2020a; Zhou et al., 2021). Compared with pure inorganic perovskites, OIHPs show much flexible structural tunability due to the introduction of organic materials (Wang et al., 2021; Shao et al., 2022). More organic cations can be used as an ideal substitute for site A when the dimension of OIHPs is reduced from 3D to 2D due to the larger tolerance factor of the 2D compound (Li et al., 2019; Yang et al., 2019; Shi et al., 2020a; Zhang et al., 2020).

In principle, replacing the B site with two different metals will produce a double perovskite structure; this kind of double perovskite-like compound can provide higher structural variability and richer physical–chemical connotations, leaving great opportunities for multifunctional materials (Shi et al., 2020a; Ma et al., 2021). Much research interest focuses on exploring molecular-based multifunctional materials (Mao et al., 2019; Zhang et al., 2021). The versatility of the material depends on the coordination of the metal (Pan et al., 2000; Zhang et al., 2013; Som et al., 2014; Kim et al., 2015; Huang et al., 2017; Li B. et al., 2018; Chen et al., 2019; Xue et al., 2020), for example, rare-earth ions have rich coordination modes and unique photoluminescence and magnetic properties because of the characteristics of their electronic configuration. They are used as one of the metals to assemble double perovskites to easily form multifunctional materials, which makes them a good candidate for the construction of multifunctional bimetallic perovskite materials (Zhang et al., 2021).

Compared with organic–inorganic hybrid halide perovskites developed rapidly with halogen atoms as the bridging ligand, there are few reports on the synthesis of double perovskite structure compounds using multi-atom nitrate as the bridging ligand (Xu et al., 2016). In 2020, we reported on a family of rare-earth double perovskite compounds, in which ferroelectric, piezoelectric, and fluorescent properties were successfully achieved (Shi et al., 2020a; Shi et al., 2020b; Hua et al., 2020). Inspired by these works, two new rare-earth alkali metal double perovskite-type compounds were synthesized by introducing rare-earth ions into the B site of double perovskite and using NO_3_^−^ as the bridging ligand: (R3HQ)_4_CsSm(NO_3_)_8_ (**1**) and (R3HQ)_4_CsEu(NO_3_)_8_ (**2**) (R3HQ = (*R*)-(-)-3-quinuclidinol). The compounds not only undergo a reversible structural phase transition at about 379 K for **1** and 375 K for **2** but the phase temperature is also increased by about 30 K compared to the reported (R3HQ)_4_RbSm(NO_3_)_8_ and (R3HQ)_4_RbSm(NO_3_)_8_. Interestingly, **1** and **2** exhibit photoluminescence under UV excitation, and the quantum yield of **2** reached 14.6%. In addition, the direct current (DC) magnetic susceptibility of compounds shows obvious paramagnetic signals. The successful construction of the compounds provides a new idea for further enriching the rare-earth double perovskite system (Zhang et al., 2020). The fast switchable dielectric behaviors, and photoluminescence and magnetic properties make them an ideal candidate for multifunctional materials (Rok et al., 2019).

## Experimental

### Synthesis of (R3HQ)_4_CsSm(NO_3_)_8_ (1) and (R3HQ)_4_CsEu(NO_3_)_8_ (2)

All reagents were purchased commercially and used as received without any further purification. First, an unseparated R3HQNO_3_ (R3HQ = (*R*)-(-)-3-quinuclidinol) aqueous solution was obtained by adding R3HQ and HNO_3_ solution (65–68%) to an aqueous solution in a molar ratio of 1:1. Then, high-quality colorless transparent block crystals were obtained by slowly evaporating the mixed aqueous solution of Sm(NO_3_)_3_/Eu(NO_3_)_3_, CsNO_3_, and R3HQNO_3_ in a molar ratio of 1:1:2 at room temperature for about 2 weeks. Anal. (%) Calc. for C_28_H_56_CsSmN_12_O_28_: C, 25.25, H, 4.23, N, 13.94. Found: C, 26.03, H, 4.37, N, 13.01. Anal. (%) Calc. for C_28_H_56_CsEuN_12_O_28_: C, 25.30, H, 4.19, N, 13.17. Found: C, 26.00, H, 4.36, N, 12.99.

### General measurements

DSC measurements were performed with a NETZSCH differential scanning calorimeter 214 Polyma in the temperature range 300–390 K under atmospheric pressure with a 20 K min^−1^ heating/cooling rate by heating and cooling the crystalline samples. The real part *ε*′ of the complex permittivity which is related to the temperature was performed using a TH2828A variable temperature dielectric measuring instrument in the temperature range of about 345–390 K. The variable temperature powder X-ray diffraction data were measured on a Rigaku D/MAX 2000 PC X-ray diffraction system with Cu-K*α* radiation in the 2*θ* range of 5°–50° with a step size of 0.02° and in the temperature range 298–403 K. Photoluminescence was recorded on an Edinburgh FL980 UV/V/NIR fluorescence spectrometer. The quantum yield was measured by using an Edinburgh FLS 1000 UV/V/NIR fluorescence spectrometer. The single-crystal X-ray diffraction data were collected on a Rigaku Synergy apparatus with graphite-monochromate Mo-Kα radiation at 278 K for **1** and 301 K for **2**, respectively. Thermogravimetric analysis was performed on NETZSCH STA 449F3 in the temperature range of about 298–1070 K.

## Results and discussion

### Crystal structure

**FIGURE 1:**
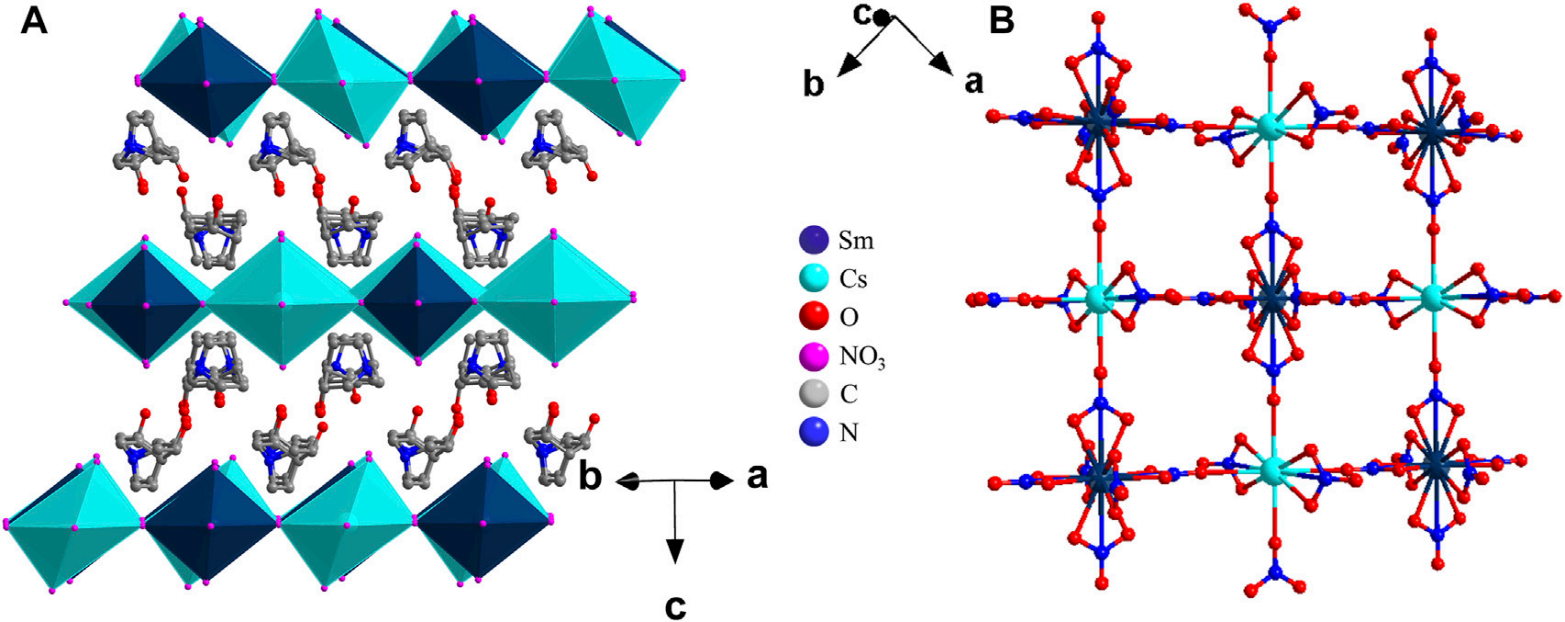
Structure packing diagrams **(A)** and the coordination and connection of atoms of an inorganic anion skeleton layer **(B)** of **1** at 278 K.

Single crystal X-ray diffraction studies revealed that **1** crystallized in the tetragonal chiral space group *P*4_3_22 (no. 95) at 278 K with cell parameter *a* = 10.1274(1) Å, *b* = 10.1274(1) Å, *c* = 46.2252(11) Å, and *Z* = 4. The crystallographic asymmetric unit of **1** comprises Sm and Cs sharing a nitrate and each connecting a complete nitrate and an incomplete nitrate, and two separate organic cations, the complete molecules being generated by inversion symmetry. Both Sm and Cs are coordinated with six NO_3_^−^ as bridging ligands to chelate into an alternately arranged corner-sharing octahedral structure to form a framework of inorganic metal layers, and the double layer of R3HQ organic cations occupied the space between the inorganic layers (Figure 1). The distance between the two adjacent inorganic metal layers is about 11.56 Å, where the distance between Sm–Sm or Cs–Cs and Sm–Cs is about 12.78 Å and 12.62 Å, respectively. The distance between Sm–Cs in the same inorganic metal layer is 7.16 Å. Therefore, the resulting octahedron is slightly distorted rather than the standard regular octahedron. The organic cation layer and inorganic metal framework layer are interleaved along the *c*-axis, and there are four inorganic layers in one cell. All C-O bonds in the organic layer are arranged along the *c*-axis, therefore, forming an infinite octahedron corner-sharing A_4_B^I^B^III^X_8_-type two-dimensional layered organic–inorganic hybrid double-metal perovskite structure. The structure of **2** is similar to that of **1** and is not described in detail here (Supplementary Figure S1).

**FIGURE 2:**
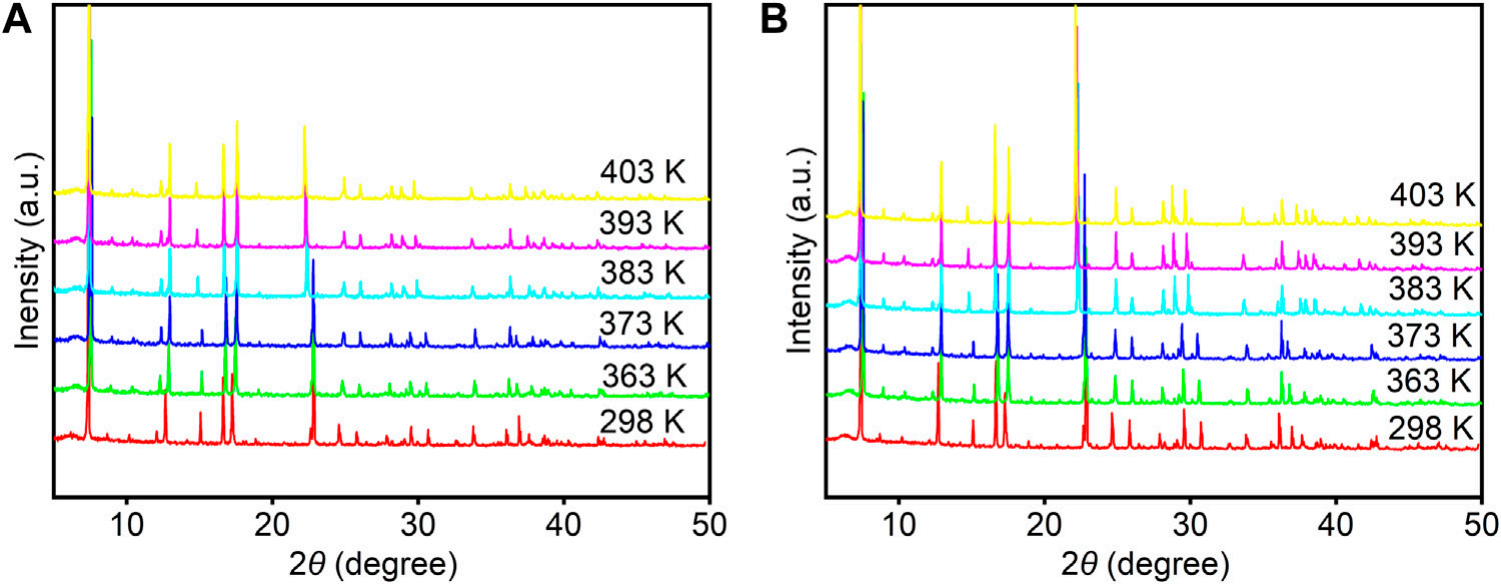
Variable temperature PXRD patterns of **1 (A)** and **2 (B)**.

We first carried out powder X-ray diffraction (PXRD) of **1** and **2** at 298 K (Supplementary Figure S2). It can be seen that the as-synthesized samples are a single pure phase, and all subsequent analyses and measurements are based on this premise. Preliminary thermal analysis has shown that the two compounds undergo a reversible structural phase transition. To further reveal the cause of the phase transition of these compounds, variable temperature X-ray single-crystal diffraction was carried out. When the temperature was above the phase transition temperature, we were unable to obtain X-ray single-crystal diffraction data because of their poor crystal quality. It is speculated that the crystal structure changes dramatically, which is caused by the excessive latent heat during the phase transition (Tang Y.-Z. et al., 2015; Ye et al., 2016; Harada et al., 2018; Yang et al., 2019; Xu et al., 2021). Therefore, variable temperature PXRD measurements were performed to determine whether the structures of **1** and **2** changed as the temperature increased. The PXRD patterns of **1** and **2** (Figures 2A,B, Supplementary Figure S3) remain unchanged in the temperature range from 298– 373 K. However, the diffraction peak of the PXRD patterns appear to change including shift, disappear, increase, and even the distance between part diffraction peaks changes obviously when the temperature continues to rise 383 K above the phase transition temperature, indicating that the crystal structure has changed because of the temperature change. The results are consistent with DSC and dielectric behavior analysis.

### Phase transition

**FIGURE 3:**
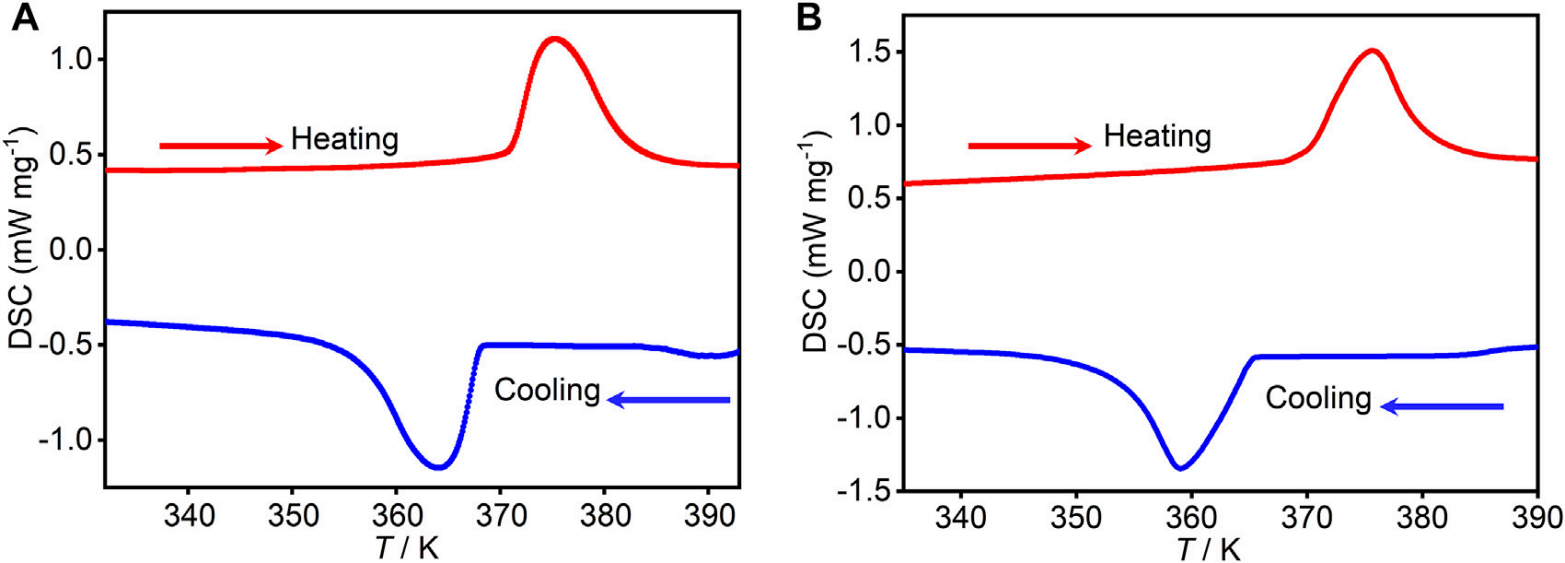
DSC curves of **1 (A)** and **2 (B)** measured under nitrogen and atmospheric pressure at a heating/cooling rate of 20 K min^−1^ at a temperature range of 330–400 K.

Differential scanning calorimetry (DSC) is an effective thermodynamic method used for determining whether crystals undergo phase transition resulting from temperature induction. The phase transitions of **1** and **2** were investigated by DSC under nitrogen and atmospheric pressure with a heating/cooling rate of 20 K min⁻^1^. The DSC curve (Figure 3A) of **1** displays an obvious thermal anomaly peak at 379 K upon heating, and the corresponding thermal anomaly peak appeared at 358 K upon cooling. Compared with the reported (R3HQ)_4_RbSm(NO_3_)_8_ and (R3HQ)_4_RbSm(NO_3_)_8_, the phase transition temperature is increased by about 30 K. Its average temperature is taken and calculated, and the change in entropy and enthalpy is 17.44 kJ mol⁻^1^ and 46.0254 J mol⁻^1^ K⁻^1^, respectively. Similarly, compound **2** showed corresponding thermal anomaly peaks at 375 and 359 K (Figure 3B). The change in entropy and enthalpy is 21.02 kJ mol⁻^1^ and 56.0608 J mol⁻^1^ K⁻^1^, respectively. That is, the change of rare-earth elements in adjacent positions has little thermal change for compounds with the same configuration. According to the Boltzmann equation, ∆*S* = R ln(*N*), where R is the gas constant and *N* is the ration of the number of respective geometrically distinguishable orientations in high- and low-temperature phases (Zhao et al., 2019), the values of *N*_1_ and *N*_2_ are calculated as 1.0043 and 1.0052, respectively. The small *N* values indicated the complex phase transition mechanism (Fu et al., 2012; Sun et al., 2012; Tang Y. et al., 2015; Sun et al., 2015; Li S.-G. et al., 2018). Combining the obvious thermal hysteresis of about 21 and 16 K during the heating–cooling cycles and wide peak patterns revealed obvious first-order phase transition characteristics. Moreover, according to the thermogravimetric (TG) curves, the phase transition temperature was significantly lower than its decomposition temperature of **1** and **2** (Supplementary Figure S4), which also confirmed that the phase transition was not caused by the decomposition of compounds.

### Dielectric properties

**FIGURE 4:**
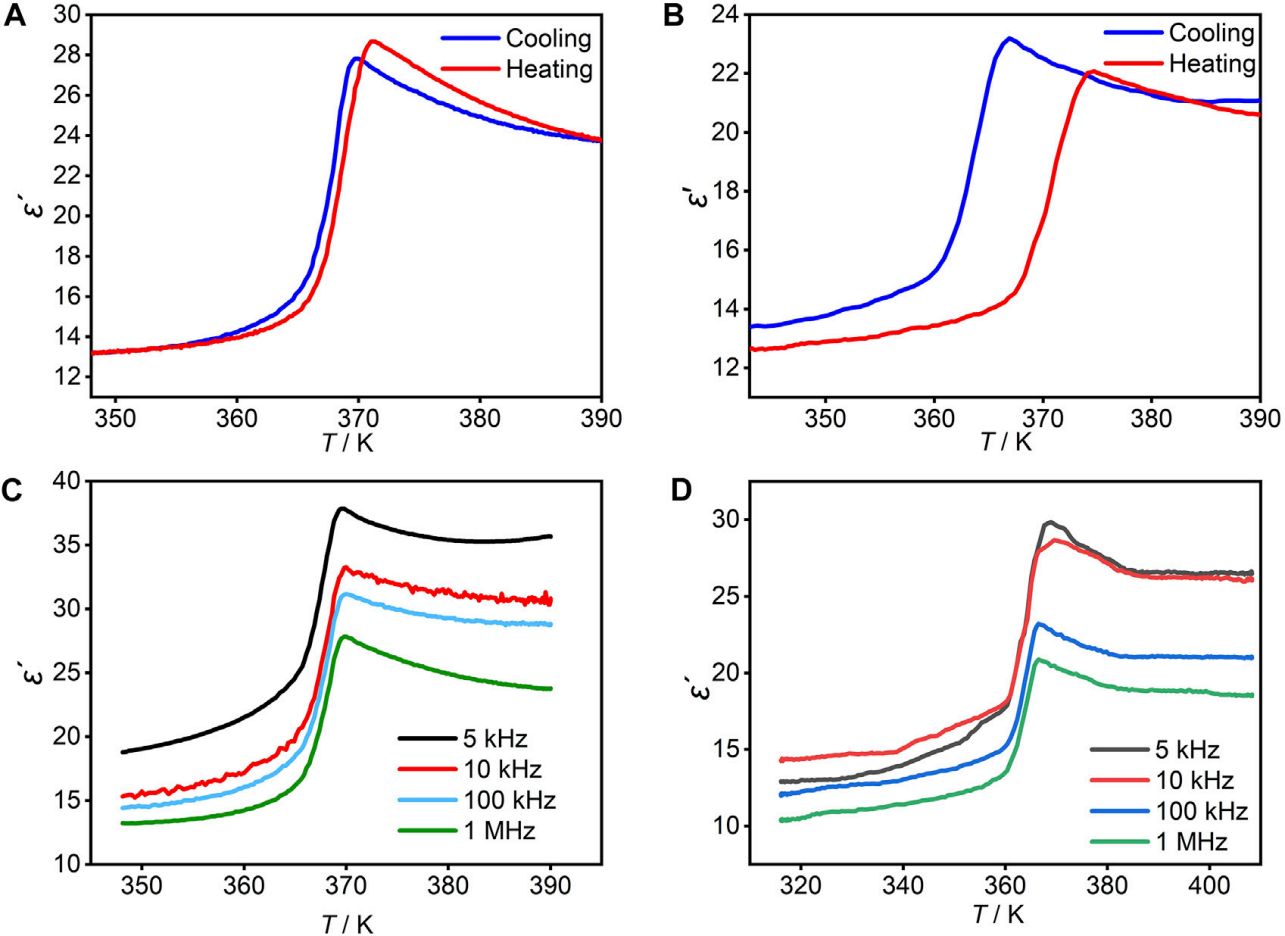
Temperature-dependence of the dielectric constant real part (*ε′*) of **1 (A)** at 100 kHz and **2 (B)** at 1 MHz with powder samples, respectively. Temperature-dependence of the dielectric constant real part (*ε′*) of **1 (C)** and **2 (D)** during heating at different frequencies.

The dielectric constant is the most basic parameter to measure dielectric materials (Fu et al., 2008a; Fu et al., 2008b; c). The real part (*ε*′) of the temperature-dependent dielectric constant of **1** was investigated in the temperature range of 348–390 K and the frequency of 100 kHz. The dielectric constant shows a trend of slow increase in the range of about 348–365 K, and a trend of slow decrease in the temperature range of about 369–395 K (Figure 4A). However, the dielectric constant changes sharply by about 2-fold in these two temperature ranges. An obvious “step-like” anomaly appeared at 371 K upon the heating process, and the corresponding anomaly appeared at 369.7 K upon the cooling process. Similarly, it can be seen that in the temperature range of 345–395 K and the frequency of 1 MHz, the real part of the dielectric constant of **2** also shows a trend of slow increase first, then a sharp rise by about 2-fold, and finally, slow decline (Figure 4B). In the heating process, there is an obvious “step-like” abnormity at 374.7 K, and corresponding steps appear at 366.8 K in the cooling process. Reversible dielectric anomalies during the heating–cooling cycle confirm the reversible phase transition of the compounds. That is, the real part of the dielectric constant of the compounds can be adjusted between the high and low dielectric states, indicating that the compounds are a good candidate for switching dielectric materials in response to thermal stimuli. This reversible transition between high and low dielectric states confirms DSC measurements that the compounds undergo a first-order reversible phase transition. In addition, it can be seen that the permittivity of the compounds varies with frequency at the same temperature, with an obvious frequency dependence (Figure 4C, Figure 4D). This is due to the effect of the external electric field, and the polarization of the dielectric cannot keep up with the change in the alternating electric field when the frequency increases, which causes dielectric loss (Gridnev and Trukhachev, 2013; Khanchaitit et al., 2013).

### Photoluminescence properties

**FIGURE 5:**
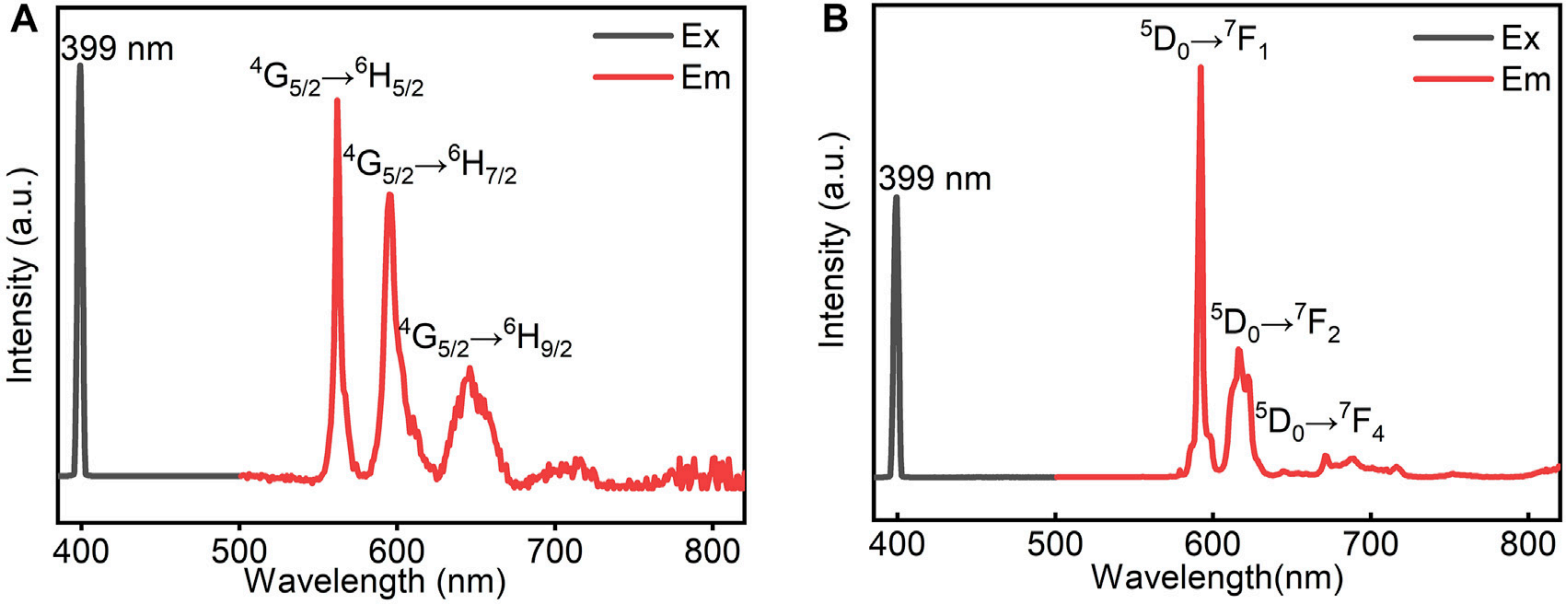
Excitation spectra and emission spectra of **1 (A)** and **2 (B)**.

One of the purposes of introducing rare-earth ions to construct a double metal perovskite structure is to combine the photoluminescence properties of rare-earth ions. Photoluminescence is caused by electron transitions of unpaired 4*f* electrons. In particular, **2** showed a distinct orange light visible to the naked eye under UV light. As shown in Figure 5A, there are three strong absorption peaks at 562, 596, and 646 nm in the emission spectrum of **1**, which are caused by the ^4^G_5/2_→^6^H_5/2_, ^4^G_5/2_→^6^H_7/2_, and ^4^G_5/2_→^6^H_9/2_ transitions of Sm^III^, respectively. Similarly, three strong absorption peaks and one weak absorption were observed at 592, 616, and 671 nm in the emission spectrum of **2** (Figure 5B), which were caused by the ^5^D_0_→^7^F_1_, ^5^D_0_→^7^F_2_, and the relatively weak ^5^D_0_→^7^F_4_ transitions of Eu^III^, respectively. Among them, the magnetic dipole transition ^5^D_0_→^7^F_1_ is obviously stronger than the electric dipole transition ^5^D_0_→^7^F_2_ because Eu^III^ is in an almost centrally symmetric position. The fluorescence quantum yield of **2** reached 14.6%. The solid-state optical absorption spectra of **1** and **2** were measured by the diffuse reflectance technique (Supplementary Figure S5). The band gaps, estimated from the absorption edges, are 3.71 eV for **1** and 3.81 eV for **2**, which are in agreement with their crystal colors.

### Magnetic properties

**FIGURE 6:**
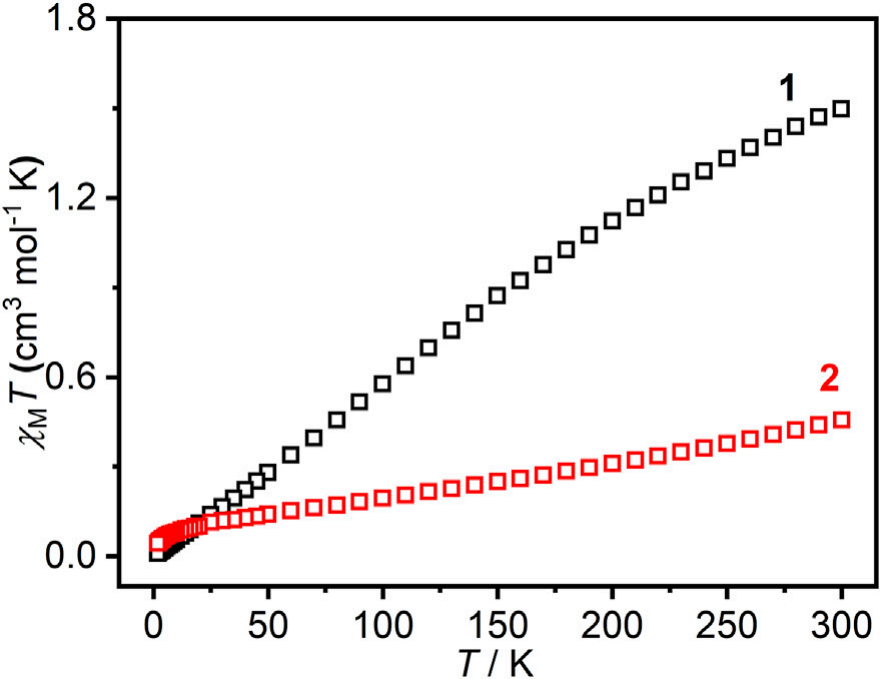
DC magnetic susceptibility of compounds **1** and **2** at the temperature range of 3–300 K and the magnetic field of 1.0 kOe.

The direct current (DC) magnetic susceptibility of compounds **1** and **2** was measured in the temperature range of 2–300 K under 1.0 kOe with powder samples (Figure 6). In general, the value of χ_m_T is zero for Eu^III^ due to *J* = 0, but an obvious paramagnetic signal was observed from the experimental data. This is because the first excited state of Eu^III^ is too close to the ground state (300 cm^−1^), which leads to the population of excited states. The ground state energy level is split into seven ^7^ *F*_J_ states caused by spin-orbit coupling. Hence, *J* is no longer 0, it can change from 0 to 6. Therefore, the value of χ_m_T gradually decreases and approaches 0 with the decrease in temperature. For **2**, Sm^III^ is the same as Eu^III^, the value of χ_m_T decreases gradually with the decrease in temperature, which is a paramagnetic signal.

## Conclusion

In summary, we obtained two organic–inorganic hybrid rare-earth double perovskites, which adopt a Ruddlesden–Popper (RP)-type perovskite structure. The reversible dielectric phase transition and switchable dielectric constant between high- and low-dielectric states of the compounds are verified by measuring temperature-dependence of the real part of the dielectric constant using differential scanning calorimetry (DSC) and variable temperature PXRD. Simultaneously, the compounds also have significant photoluminescence and paramagnetic behavior. Therefore, the compounds have potential application in the field of stimulus-responsive multifunctional intelligent materials, and the successful synthesis of the two compounds further enriches the rare-earth two-dimensional perovskite family.

## Data Availability

The raw data supporting the conclusion of this article will be made available by the authors, without undue reservation.
