# Supplementary material for: Synthesis, dielectric, magnetic, and photoluminescence properties of two new hybrid rare-earth double perovskites
Source: Front Chem. 2022 Aug 5;10:969156. doi: 10.3389/fchem.2022.969156 (PMC9389020; doi:10.3389/fchem.2022.969156)

checkCIF/PLATON report

Structure factors have been supplied for datablock(s) WQW190813_4_RT

THIS REPORT IS FOR GUIDANCE ONLY. IF USED AS PART OF A REVIEW PROCEDURE FOR PUBLICATION, IT SHOULD NOT REPLACE THE EXPERTISE OF AN EXPERIENCED CRYSTALLOGRAPHIC REFEREE.

No syntax errors found. CIF dictionary Interpreting this report

Datablock: WQW190813_4_RT

Bond precision: C-C = 0.0137 A Wavelength=0.71073

Cell:	a=10.1274(1)	b=10.1274(1)	c=46.2252(11)
	alpha=90	beta=90	gamma=90
Temperature:	278 K		

	Calculated	Reported
Volume	4741.05(15)	4741.05(13)
Space group	P 43 2 2	P 43 2 2
Hall group	P 4cw 2c	P 4cw 2c
Moiety formula	Cs N8 O24 Sm, 4(C7 H14 N O)	Cs N8 O24 Sm, 4(C7 H14 N O)
Sum formula	C28 H56 Cs N12 O28 Sm	C28 H56 Cs N12 O28 Sm
Mr	1292.12	1292.09
Dx,g cm-3	1.810	1.810
Z	4	4
Mu (mm-1)	2.099	2.099
F000	2596.0	2597.9
F000’	2595.25	
h,k,lmax	15,15,68	14,9,65
Nref	8085[4750]	7028
Tmin,Tmax	0.664,0.657	0.767,1.000
Tmin’	0.651	

Correction method= # Reported T Limits: Tmin=0.767 Tmax=1.000
AbsCorr = MULTI-SCAN

Data completeness= 1.48/0.87 Theta(max)= 31.790

R(reflections)= 0.0504(5674) wR2(reflections)= 0.1368(7028)

S = 1.026 Npar= 321

The following ALERTS were generated. Each ALERT has the format
test-name_ALERT_alert-type_alert-level.
Click on the hyperlinks for more details of the test.

Alert level C

```
PLAT068_ALERT_1_C Reported F000 Differs from Calcd (or Missing)...     Please Check
PLAT230_ALERT_2_C Hirshfeld Test Diff for    O2       --N1       .        6.5 s.u.
PLAT230_ALERT_2_C Hirshfeld Test Diff for    O5       --N2       .        6.7 s.u.
PLAT234_ALERT_4_C Large Hirshfeld Difference N6       --C1       .       0.16 Ang.
PLAT234_ALERT_4_C Large Hirshfeld Difference C1       --C2       .       0.21 Ang.
PLAT241_ALERT_2_C High   'MainMol' Ueq as Compared to Neighbors of        O3 Check
PLAT241_ALERT_2_C High   'MainMol' Ueq as Compared to Neighbors of        O9 Check
PLAT241_ALERT_2_C High   'MainMol' Ueq as Compared to Neighbors of       O11 Check
PLAT242_ALERT_2_C Low    'MainMol' Ueq as Compared to Neighbors of       Sm1 Check
PLAT242_ALERT_2_C Low    'MainMol' Ueq as Compared to Neighbors of       Cs1 Check
PLAT242_ALERT_2_C Low    'MainMol' Ueq as Compared to Neighbors of        N4 Check
PLAT243_ALERT_4_C High   'Solvent' Ueq as Compared to Neighbors of        C1 Check
PLAT243_ALERT_4_C High   'Solvent' Ueq as Compared to Neighbors of        C7 Check
PLAT243_ALERT_4_C High   'Solvent' Ueq as Compared to Neighbors of        C8 Check
PLAT243_ALERT_4_C High   'Solvent' Ueq as Compared to Neighbors of        C9 Check
PLAT244_ALERT_4_C Low    'Solvent' Ueq as Compared to Neighbors of        N6 Check
PLAT244_ALERT_4_C Low    'Solvent' Ueq as Compared to Neighbors of        C6 Check
PLAT244_ALERT_4_C Low    'Solvent' Ueq as Compared to Neighbors of        N7 Check
PLAT244_ALERT_4_C Low    'Solvent' Ueq as Compared to Neighbors of       C11 Check
PLAT250_ALERT_2_C Large U3/U1 Ratio for Average U(i,j) Tensor ....        2.1 Note
PLAT342_ALERT_3_C Low Bond Precision on  C-C Bonds ...............    0.01367 Ang.
PLAT972_ALERT_2_C Check Calcd Resid. Dens.  0.44Ang From O13            -2.03 eA-3
PLAT972_ALERT_2_C Check Calcd Resid. Dens.  0.53Ang From O8             -1.97 eA-3
PLAT972_ALERT_2_C Check Calcd Resid. Dens.  0.44Ang From O8             -1.78 eA-3
PLAT972_ALERT_2_C Check Calcd Resid. Dens.  0.75Ang From Sm1            -1.67 eA-3
PLAT972_ALERT_2_C Check Calcd Resid. Dens.  0.56Ang From N3             -1.54 eA-3
PLAT973_ALERT_2_C Check Calcd Positive Resid. Density on       Sm1       1.12 eA-3
PLAT975_ALERT_2_C Check Calcd Resid. Dens.  0.73Ang From O15     .       0.62 eA-3
PLAT977_ALERT_2_C Check Negative Difference Density on H12A      .      -0.31 eA-3
```

Alert level G

```
PLAT002_ALERT_2_G Number of Distance or Angle Restraints on AtSite          2 Note
PLAT003_ALERT_2_G Number of Uiso or Uij Restrained non-H Atoms ...         22 Report
PLAT004_ALERT_5_G Polymeric Structure Found with Maximum Dimension          2 Info
PLAT073_ALERT_1_G H-atoms ref, but _hydrogen_treatment Reported as     constr Check
PLAT083_ALERT_2_G SHELXL Second Parameter in WGHT  Unusually Large      12.34 Why ?
PLAT152_ALERT_1_G The Supplied and Calc. Volume s.u. Differ by ...          2 Units
PLAT172_ALERT_4_G The CIF-Embedded .res File Contains DFIX Records          2 Report
PLAT177_ALERT_4_G The CIF-Embedded .res File Contains DELU Records          1 Report
PLAT178_ALERT_4_G The CIF-Embedded .res File Contains SIMU Records         13 Report
PLAT186_ALERT_4_G The CIF-Embedded .res File Contains ISOR Records          3 Report
PLAT232_ALERT_2_G Hirshfeld Test Diff (M-X)  Sm1      --O1       .       10.1 s.u.
PLAT232_ALERT_2_G Hirshfeld Test Diff (M-X)  Sm1      --O3       .        8.9 s.u.
PLAT232_ALERT_2_G Hirshfeld Test Diff (M-X)  Sm1      --O4       .       12.6 s.u.
PLAT232_ALERT_2_G Hirshfeld Test Diff (M-X)  Sm1      --O6       .       11.2 s.u.
PLAT232_ALERT_2_G Hirshfeld Test Diff (M-X)  Sm1      --O7       .       20.0 s.u.
PLAT769_ALERT_4_G CIF Embedded explicitly supplied scattering data     Please Note
PLAT794_ALERT_5_G Tentative Bond Valency for Sm1        (III)      .       3.94 Info
PLAT860_ALERT_3_G Number of Least-Squares Restraints .............        140 Note
```

```
PLAT910_ALERT_3_G Missing # of FCF Reflection(s) Below Theta(Min).          1 Note
PLAT912_ALERT_4_G Missing # of FCF Reflections Above STh/L=  0.600        466 Note
PLAT933_ALERT_2_G Number of HKL-OMIT Records in Embedded .res File          1 Note
PLAT958_ALERT_1_G Calculated (ThMax) and Actual (FCF) Lmax Differ.          3 Units
PLAT978_ALERT_2_G Number C-C Bonds with Positive Residual Density.          0 Info
PLAT982_ALERT_1_G The Cs-f'=   -0.2534 Deviates from  IT-value =      -0.3680 Check
PLAT982_ALERT_1_G The Sm-f'=    0.0751 Deviates from  IT-value =      -0.1638 Check
PLAT983_ALERT_1_G The Cs-f"=    2.1896 Deviates from  IT-Value =       2.1192 Check
PLAT983_ALERT_1_G The Sm-f"=    3.7280 Deviates from  IT-Value =       3.4418 Check
```

```
   0 ALERT level A = Most likely a serious problem - resolve or explain
   0 ALERT level B = A potentially serious problem, consider carefully
  29 ALERT level C = Check. Ensure it is not caused by an omission or oversight
  27 ALERT level G = General information/check it is not something unexpected

   8 ALERT type 1 CIF construction/syntax error, inconsistent or missing data
  27 ALERT type 2 Indicator that the structure model may be wrong or deficient
   3 ALERT type 3 Indicator that the structure quality may be low
  16 ALERT type 4 Improvement, methodology, query or suggestion
   2 ALERT type 5 Informative message, check
```

It is advisable to attempt to resolve as many as possible of the alerts in all categories. Often the minor alerts point to easily fixed oversights, errors and omissions in your CIF or refinement strategy, so attention to these fine details can be worthwhile. In order to resolve some of the more serious problems it may be necessary to carry out additional measurements or structure refinements. However, the purpose of your study may justify the reported deviations and the more serious of these should normally be commented upon in the discussion or experimental section of a paper or in the "special_details" fields of the CIF. checkCIF was carefully designed to identify outliers and unusual parameters, but every test has its limitations and alerts that are not important in a particular case may appear. Conversely, the absence of alerts does not guarantee there are no aspects of the results needing attention. It is up to the individual to critically assess their own results and, if necessary, seek expert advice.

Publication of your CIF in IUCr journals

A basic structural check has been run on your CIF. These basic checks will be run on all CIFs submitted for publication in IUCr journals (*Acta Crystallographica*, *Journal of Applied Crystallography*, *Journal of Synchrotron Radiation*); however, if you intend to submit to *Acta Crystallographica Section C* or *E* or *IUCrData*, you should make sure that full publication checks are run on the final version of your CIF prior to submission.

Publication of your CIF in other journals

Please refer to the *Notes for Authors* of the relevant journal for any special instructions relating to CIF submission.

PLATON version of 18/05/2022; check.def file version of 17/05/2022

Datablock WQW190813_4_RT - ellipsoid plot

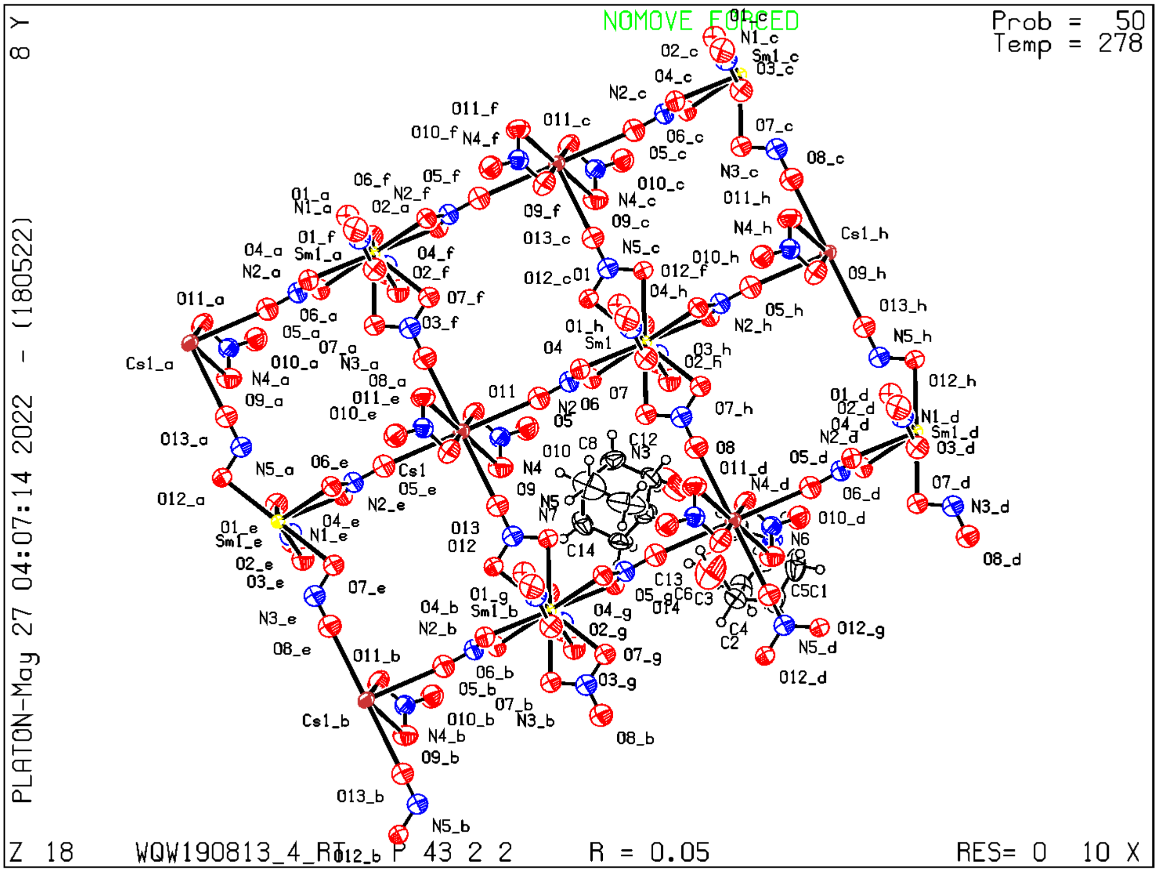

Supplement: Supplementary file 1 [file DataSheet1.ZIP › Cif and CheckCif/CheckCif_Compound 1.pdf]
