# Supplementary material for: Synthesis, dielectric, magnetic, and photoluminescence properties of two new hybrid rare-earth double perovskites
Source: Front Chem. 2022 Aug 5;10:969156. doi: 10.3389/fchem.2022.969156 (PMC9389020; doi:10.3389/fchem.2022.969156)

checkCIF/PLATON report

Structure factors have been supplied for datablock(s) zj210825_1h_rt

THIS REPORT IS FOR GUIDANCE ONLY. IF USED AS PART OF A REVIEW PROCEDURE FOR PUBLICATION, IT SHOULD NOT REPLACE THE EXPERTISE OF AN EXPERIENCED CRYSTALLOGRAPHIC REFEREE.

No syntax errors found. CIF dictionary Interpreting this report

Datablock: zj210825_1h_rt

```
Bond precision:   C-C = 0.0200 A          Wavelength=0.71073

Cell:               a=10.1157(3)      b=10.1157(3)      c=46.210(2)
                    alpha=90          beta=90           gamma=90
Temperature:        302 K

                  Calculated                        Reported
Volume            4728.6(3)                         4728.5(3)
Space group       P 43 2 2                          P 43 2 2
Hall group        P 4cw 2c                          P 4cw 2c
Moiety formula    Cs Eu N8 O24, 4(C7 H14 N O) Cs Eu N8 O24, 4(C7 H14 N O)
Sum formula       C28 H56 Cs Eu N12 O28             C28 H56 Cs Eu N12 O28
Mr                1293.73                           1293.71
Dx,g cm-3         1.817                             1.817
Z                 4                                 4
Mu (mm-1)         2.189                             2.189
F000              2600.0                            2600.0
F000'             2599.28
h,k,lmax          12,12,57                          12,11,57
Nref              4669[ 2819]                       4661
Tmin,Tmax         0.652,0.803                       0.833,1.000
Tmin'             0.639

Correction method= # Reported T Limits: Tmin=0.833 Tmax=1.000
AbsCorr = MULTI-SCAN

Data completeness= 1.65/1.00       Theta(max)= 25.999

                                                      wR2(reflections)=
R(reflections)= 0.0533( 3849)                         0.1463( 4661)

S = 1.056                  Npar= 320
```

The following ALERTS were generated. Each ALERT has the format
test-name_ALERT_alert-type_alert-level.
Click on the hyperlinks for more details of the test.

Alert level C

```
PLAT241_ALERT_2_C High   'MainMol' Ueq as Compared to Neighbors of          O3 Check
PLAT241_ALERT_2_C High   'MainMol' Ueq as Compared to Neighbors of          O9 Check
PLAT241_ALERT_2_C High   'MainMol' Ueq as Compared to Neighbors of         O11 Check
PLAT242_ALERT_2_C Low    'MainMol' Ueq as Compared to Neighbors of         Eu1 Check
PLAT242_ALERT_2_C Low    'MainMol' Ueq as Compared to Neighbors of         Cs1 Check
PLAT242_ALERT_2_C Low    'MainMol' Ueq as Compared to Neighbors of          N4 Check
PLAT244_ALERT_4_C Low    'Solvent' Ueq as Compared to Neighbors of          N6 Check
PLAT244_ALERT_4_C Low    'Solvent' Ueq as Compared to Neighbors of          C6 Check
PLAT244_ALERT_4_C Low    'Solvent' Ueq as Compared to Neighbors of         C13 Check
PLAT342_ALERT_3_C Low Bond Precision on  C-C Bonds ...............        0.02 Ang.
PLAT360_ALERT_2_C Short  C(sp3)-C(sp3) Bond  C11      - C12      .        1.40 Ang.
PLAT972_ALERT_2_C Check Calcd Resid. Dens.  0.49Ang From O8              -1.84 eA-3
PLAT972_ALERT_2_C Check Calcd Resid. Dens.  0.45Ang From O13             -1.58 eA-3
PLAT976_ALERT_2_C Check Calcd Resid. Dens.  0.59Ang From O5      .       -0.55 eA-3
```

Alert level G

```
PLAT003_ALERT_2_G Number of Uiso or Uij Restrained non-H Atoms ...          29 Report
PLAT004_ALERT_5_G Polymeric Structure Found with Maximum Dimension           2 Info
PLAT007_ALERT_5_G Number of Unrefined Donor-H Atoms ..............           4 Report
PLAT083_ALERT_2_G SHELXL Second Parameter in WGHT  Unusually Large       17.92 Why ?
PLAT177_ALERT_4_G The CIF-Embedded .res File Contains DELU Records           2 Report
PLAT178_ALERT_4_G The CIF-Embedded .res File Contains SIMU Records          16 Report
PLAT186_ALERT_4_G The CIF-Embedded .res File Contains ISOR Records           6 Report
PLAT232_ALERT_2_G Hirshfeld Test Diff (M-X)  Eu1      --O4       .         9.0 s.u.
PLAT232_ALERT_2_G Hirshfeld Test Diff (M-X)  Eu1      --O6       .         7.0 s.u.
PLAT794_ALERT_5_G Tentative Bond Valency for Eu1       (III)     .        3.15 Info
PLAT860_ALERT_3_G Number of Least-Squares Restraints .............         181 Note
PLAT910_ALERT_3_G Missing # of FCF Reflection(s) Below Theta(Min).           3 Note
PLAT933_ALERT_2_G Number of HKL-OMIT Records in Embedded .res File           1 Note
PLAT955_ALERT_1_G Reported (CIF) and Actual (FCF) Lmax Differ by .           1 Units
PLAT967_ALERT_5_G Note: Two-Theta Cutoff Value in Embedded .res ..        52.0 Degree
PLAT978_ALERT_2_G Number C-C Bonds with Positive Residual Density.           1 Info
```

```
   0 ALERT level A = Most likely a serious problem - resolve or explain
   0 ALERT level B = A potentially serious problem, consider carefully
  14 ALERT level C = Check. Ensure it is not caused by an omission or oversight
  16 ALERT level G = General information/check it is not something unexpected

   1 ALERT type 1 CIF construction/syntax error, inconsistent or missing data
  16 ALERT type 2 Indicator that the structure model may be wrong or deficient
   3 ALERT type 3 Indicator that the structure quality may be low
   6 ALERT type 4 Improvement, methodology, query or suggestion
   4 ALERT type 5 Informative message, check
```

It is advisable to attempt to resolve as many as possible of the alerts in all categories. Often the minor alerts point to easily fixed oversights, errors and omissions in your CIF or refinement strategy, so attention to these fine details can be worthwhile. In order to resolve some of the more serious problems it may be necessary to carry out additional measurements or structure refinements. However, the purpose of your study may justify the reported deviations and the more serious of these should normally be commented upon in the discussion or experimental section of a paper or in the "special_details" fields of the CIF. checkCIF was carefully designed to identify outliers and unusual parameters, but every test has its limitations and alerts that are not important in a particular case may appear. Conversely, the absence of alerts does not guarantee there are no aspects of the results needing attention. It is up to the individual to critically assess their own results and, if necessary, seek expert advice.

Publication of your CIF in IUCr journals

A basic structural check has been run on your CIF. These basic checks will be run on all CIFs submitted for publication in IUCr journals (*Acta Crystallographica*, *Journal of Applied Crystallography*, *Journal of Synchrotron Radiation*); however, if you intend to submit to *Acta Crystallographica Section C* or *E* or *IUCrData*, you should make sure that full publication checks are run on the final version of your CIF prior to submission.

Publication of your CIF in other journals

Please refer to the *Notes for Authors* of the relevant journal for any special instructions relating to CIF submission.

PLATON version of 18/05/2022; check.def file version of 17/05/2022

Datablock zj210825_1h_rt - ellipsoid plot

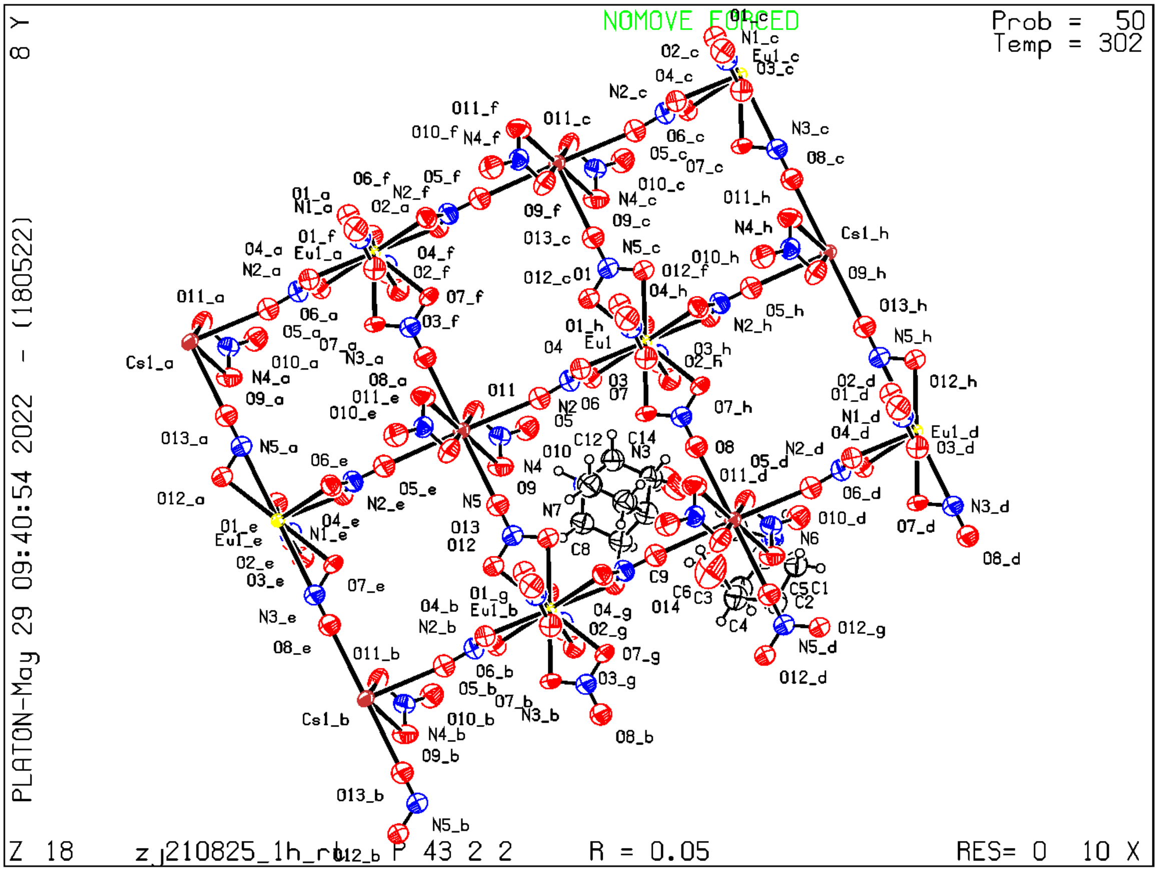

Supplement: Supplementary file 1 [file DataSheet1.ZIP › Cif and CheckCif/CheckCif_Compound 2.pdf]
