# Supplementary material for: Synthesis, dielectric, magnetic, and photoluminescence properties of two new hybrid rare-earth double perovskites
Source: Front Chem. 2022 Aug 5;10:969156. doi: 10.3389/fchem.2022.969156 (PMC9389020; doi:10.3389/fchem.2022.969156)
Supplement: Supplementary file 2 [file DataSheet2.docx]

Supporting Information

Synthesis, dielectric, magnetic and photoluminescence properties of two new hybrid rare earth double perovskites

Ze-Jie Wang,^1^ Long-He Li,^1^ Yan Feng,^1^ Qin-Wen Wang,^1^ Ling-Kun Wu,^1^ Jian-Rong Li*^1^ and Heng-Yun Ye*^1^

^1^ Chaotic Matter Science Research Center, Jiangxi University of Science and Technology, Ganzhou 341000, Jiangxi, P. R. China.

*** Correspondence:**Corresponding Author: Jian-Rong Li, Heng-Yun Ye
 [jrli@fjirsm.ac.cn](mailto:jrli@fjirsm.ac.cn), hyye@seu.edu.cn


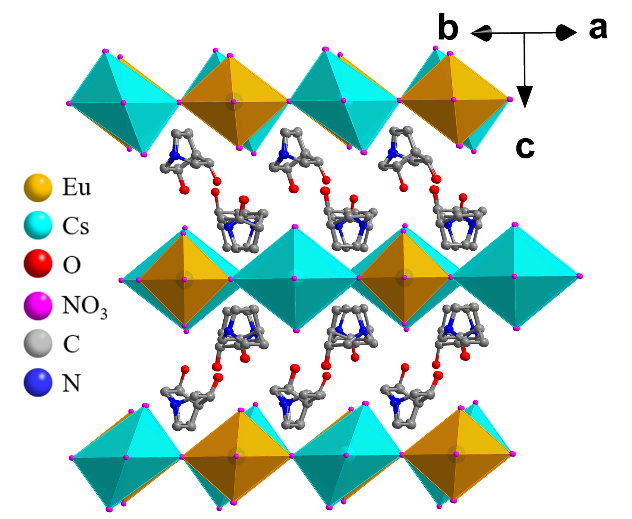


**Figure S1** Structure packing diagram of **2** at 301 K.

**
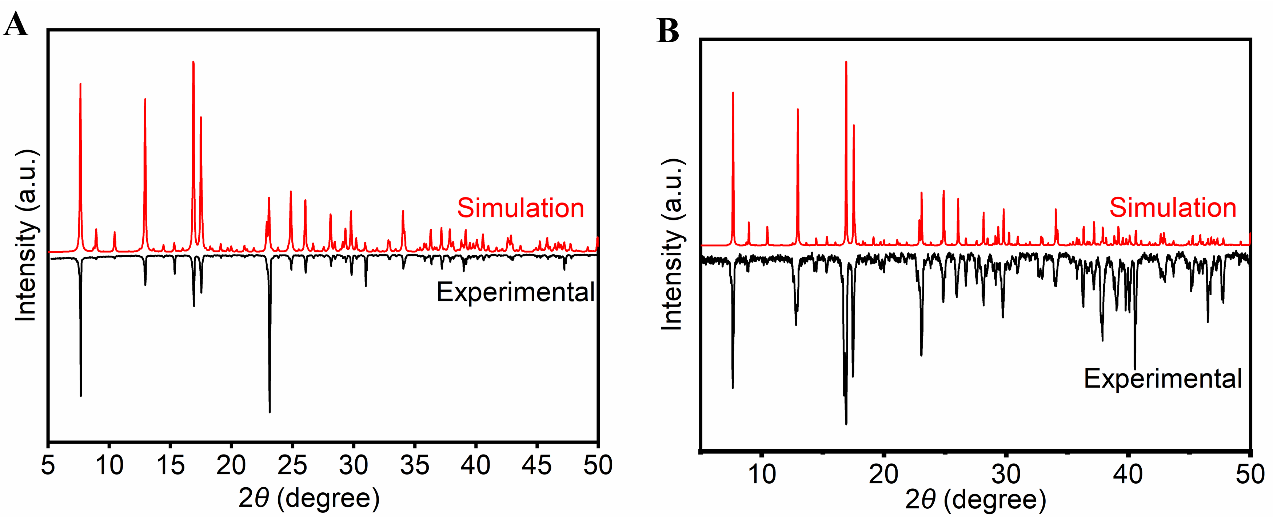
**

**Figure S2** Simulated and experimental PXRD patterns of **1 (A)** and **2 (B)** at room temperature.


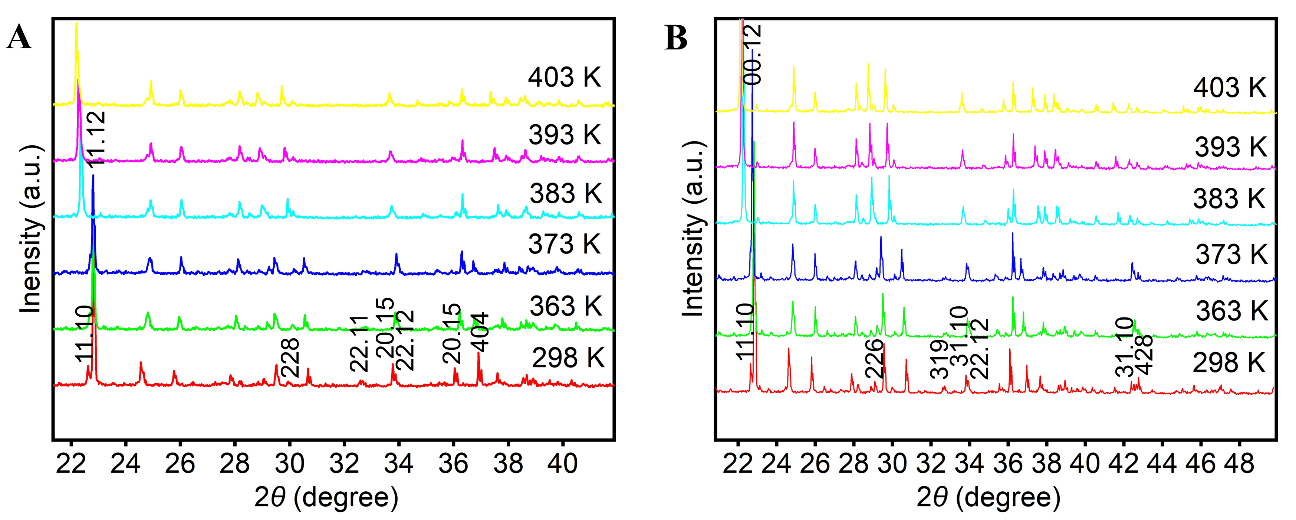


**Figure S3** Partial amplification peaks of variable temperature PXRD patterns of compounds **1** (**A**) and **2** (**B**).





**Figure S4** The TG curves of compounds **1** and **2**.

**
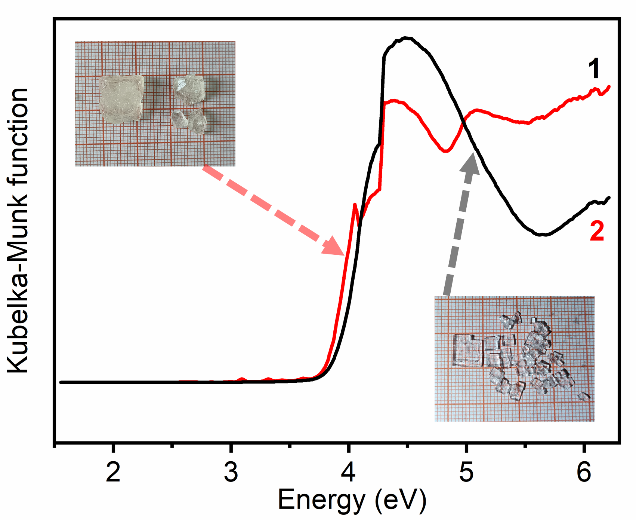
**

**Figure S5** Solid-state optical absorption spectra of **1** and **2**. Inset: crystal photos of **1** and **2**, the sizes are 13 mm × 13 mm × 6 mm and 9 mm × 9 mm × 2 mm, respectively.

**Table S1** Crystallographic data and structure refinements for **1** and **2**.

|  | Compound **1** | Compound **2** |
| --- | --- | --- |
| Formula | C_28_H_56_CsSmN_12_O_28_ | C_28_H_56_CsEuN_12_O_28_ |
| Formula weight | 1292.12 | 1293.71 |
| Temperature (K) | 278 | 301 |
| Crystal system | tetragonal | tetragonal |
| Space group | *P*4_3_22 | *P*4_3_22 |
| *a*/Å | 10.1274(1) | 10.1175(3) |
| *b*/Å | 10.1274(1) | 10.1175(3) |
| *c*/Å | 46.2252(11) | 46.210(2) |
| Volume(Å^3^) | 4741.05(13) | 4728.5(3) |
| *Z* | 4 | 4 |
| *ρ*_calc_g/cm^3^ | 1.810 | 1.817 |
| μ/mm^-1^ | 2.099 | 2.189 |
| *F*(000) | 2596.0 | 2600.0 |
| 2*θ* | 4.022 to 63.586 | 4.396 to 51.998 |
| Index ranges | -10≤*h*≤14  -13≤*k*≤12  -60≤*I*≤65 | -12≤*h*≤9  -10≤*k*≤11  -57≤*I*≤50 |
| Goodness-of-fit on *F*^2^ | 1.051 | 1.067 |
| Final *R* indexes [*I*>=2*σ* (I)] | *R*_1_ = 0.0497  *wR*_2_ = 0.1353 | *R*_1_ = 0.0489  *wR*_2_ = 0.1288 |
| Final *R* indexes [all data] | *R*1 = 0.0497  *wR*_2_ = 0.1353 | *R*_1_ = 0.0489  *wR*_2_ = 0.1288 |
| Flack parameter | 0.010(8) | 0.024(13) |
| Reflections collected | 7028[*R*_int_ = 0.0417, *R*_sigma_ = 0.0316] | 4661[*R*_int_ = 0.0446, *R*_sigma_ = 0.0439] |
| CCDC | 2174659 | 2174658 |
